# Investigation of apoptotic and autophagic effects of chronic roflumilast use on testicular tissue in rats by immunohistochemical and immunofluorescence methods

**DOI:** 10.22038/IJBMS.2023.65948.14507

**Published:** 2023-03

**Authors:** Arzu Gezer, Ebru Karadağ Sari

**Affiliations:** 1 Vocational School of Health Services, Atatürk University, Erzurum, Turkey; 2 Department of Histology and Embryology, Faculty of Veterinary Medicine, Kafkas University, Kars, Turkey

**Keywords:** Apoptosis, Autophagy, Rat, Roflumilast, Testis, Testosterone

## Abstract

**Objective(s):**

The present study aims to determine how various dosages of chronic roflumilast affect testicular tissue and testosterone levels in healthy rats.

**Materials and Methods:**

Biochemical tests, along with histopathological, immunohistochemical, and immunofluorescence studies, were carried out.

**Results:**

Loss of tissue in the seminiferous epithelium, degeneration in the interstitial area, a separation between cells, desquamation, interstitial edema, and degenerative alterations in testicular tissue were observed in roflumilast groups when compared with the other groups. While apoptosis and autophagy were statistically negligible in the control and sham groups, the roflumilast groups had significantly higher apoptotic and autophagic alterations, as well as immunopositivity. Serum testosterone levels in the 1 mg/kg roflumilast group were lower than those in the control, sham, and 0.5 mg/kg roflumilast groups.

**Conclusion:**

Analyses of the research findings revealed that continuous usage of the broad-spectrum active component roflumilast exerted unfavorable effects on the testicular tissue and testosterone levels of rats.

## Introduction

Roflumilast (ROF) is a second-generation and forcible phosphodiesterase-4 (PDE4) inhibitor (1). Phosphodiesterases (PDEs) are phosphohydrolase enzymes that cause hydrolysis of the 3’-phosphate bridges of intracellular secondary messenger molecules, cyclic guanosine monophosphate (cGMP), and cyclic adenosine monophosphate (cAMP), that is, they regulate the continuity and termination of cAMP and cGMP signals in the cell (2). As PDEs have a critical effect on signaling pathways in various pharmacological processes, including cell function, they have drawn the attention of researchers relevant to many disease pathologies (3). PDEs, which are a large family of enzymes, reportedly consist of 11 subtypes (PDE1-PDE11) and over 40 isoforms (4). 

Phosphodiesterase inhibitors such as roflumilast act by inhibiting the breakdown of intracellular cAMP, reducing inflammation, suppressing cell proliferation, cytokine production, and chemotaxis. ROF, which was used as an antidepressant in the 1980s, is the first selector PDE4 inhibitor approved for human usage in 2010, with the primary indication being chronic obstructive pulmonary disease (COPD) (5, 6). In the later years, it started being used in the fields of arthritis, neurodegenerative diseases, liver, and dermatology (7-9).

Apoptosis and autophagy are phenomena that occur in many cases as cell death mechanisms (9). Apoptosis is the mechanism in which the cell self-destructs, is regulated by genes, needs protein synthesis and energy, and maintains the balance in the organism (10). This mechanism of death is divided into two caspase-dependent and caspase-independent apoptosis. To induce programmed cell death, the Apoptosis-Inducing Factor (AIF) protein plays a role in the initiation of the caspase-independent apoptotic pathway by triggering chromatin condensation and DNA fragmentation in the cell and regulating the permeability of the mitochondrial membrane (11). Autophagy, on the other hand, is a catabolic mechanism that sends not only cytosolic proteins but also intracellular cytosolic components, organelles, and aggregates to lysosomes for degradation, preventing the accumulation of misfolded proteins and destroying unwanted organelles (12). Light Chain 3βeta (LC3B) protein, which is a reagent of autophagy, is effective in initiating the autophagic pathway (13). 

In a study in mice, ROF was administered in different doses (250, 500, and 1000 µg/kg) for 5 days by inhaler for 15 min. At the end of the experiment, it was claimed that medium and high doses of ROF were more effective in improving asthma (14). In the pharmacology review published by the FDA in 2011, it was reported that headache, gastrointestinal disorders, dizziness, palpitations, flu, and arterial hypotension were observed after a single oral application of 2.5 and 5 mg in Phase 1 studies of ROF (15).

When the studies are examined in the literature, the protective properties of ROF in different tissues have been reported against experimentally induced damages and some toxic agents. However, there is no *in vivo* scientific study that investigated healthy testicular tissue toxicity after chronic use. Chronic use of ROF, which has a widespread clinical use, was found to be apoptotic, caspase-dependent (Caspase-3), caspase-independent (AIF), autophagic, expressions of (LC3B) proteins, and their effects on rat testicular tissue were investigated by analyzing serum testosterone levels in this study.

## Materials and Methods

***Animal study***

Approval for this study was obtained from the Animal Experiments Local Ethics Committee of Atatürk University, Faculty of Veterinary Medicine, on 31.07.2019 with the ethics committee number 9/130. The rats used in the study were obtained from Atatürk University Medical Experimental Research and Application Center and the experimental part of the study was conducted in this center. The research was carried out following the principles of the Declaration of Helsinki. In the study, a total of 36 Sprague-Dawley male rats, 40 days old and weighing 180-200 g were used. The groups were randomly formed. They were fed *ad libitum* with tap water in an environment with 12 hr of darkness and 12 hr of lighting and kept in standard cages. The ROF groups were given 0.5 and 1 mg/kg ROF by oral gavage at the same time every day for 4 weeks (16)(Table 1). At the end of the experiment, deep sedation of rats was achieved with Sevoflurane (Sevorane®, Abbott Lab. Istanbul, Turkey), cervical dislocation was performed, intracardiac blood samples were obtained, and testicular tissue samples were taken. Body weights and testes weights of rats in all groups were measured. Testicular tissue samples were placed in a 10% buffered neutral formalin solution, and after routine histological procedures, they were blocked in paraffin.

***Histological studies***

Hematoxylin and Eosin (H&E) staining technique was applied to the sections taken from the blocks to examine the general structure of testicular tissue. The preparations were examined under a light microscope (Nikon BX51), 6 selected fields were chosen randomly, and the experimental groups were compared by scoring according to the Johnsen criteria to reveal the possible severity of ROF damage to the testicular seminiferous tubule cells (17, 18).

***Immunohistochemical studies***

The 5 μm sections taken from the blocks were passed through xylol and alcohol series and washed with PBS for 10 min in 3% H_2_O_2_ and treated with antigen retrieval solution. Afterward, a protein block was applied to completely cover the sections. Then the sections of Table II were incubated with the indicated primary antibodies. After applying Mouse and Rabbit Specific HRP/DAB IHC Detection Kit-Micro-polymer kit (Abcam, Catalog No. ab236466) as secondary antibody and 3-3’Diaminobenzidine (DAB) as a chromogen to tissue samples, counterstaining was performed with Mayer’s Hematoxylin. Tissue samples were passed through graded alcohols and xylol series and sealed with entellan.

***Immunofluorescence staining method***

The procedures specified in immunohistochemical staining were applied to the prepared sections until incubation with the primary antibody. The sections were then incubated at 37 ^°^C for 45 min with the primary antibodies (1/200 dilution ratio) indicated in Table 2. After washing with PBS, the sections were incubated again with the fluorescence-linked secondary antibodies in Table 3 (1/50 dilution ratio) for 45 min at 37 ^°^C in the dark. DAPI was dripped onto the tissue samples and covered with a coverslip and examined under a fluorescent microscope (Zeiss Axio scope, German). Using a fluorescence microscope, immunofluorescence findings were determined and ranked as: absent (−), mild (+), moderate (++) or severe (+++), by using the image analysis computer program named Image J 1.43, according to the intensity of staining: none (0), mild (1), moderate (2), and severe (3) (19).

***Biochemical analysis***

Serum testosterone levels were analyzed with the trademark ELISA kit (Biovision, Catalog No: K7418) according to the kit procedure. Results are given in ng/ml for testosterone.

***Histometric calculation of seminiferous tubule diameters***

To measure the diameters of the seminiferous tubules, 2 measurements were made from 10 randomly selected round and near-circular seminiferous tubules from each animal at 20x magnification, and their averages were taken (20). The measurement was made using the image analysis computer program called Image J 1.43 (21).

***Statistical analysis***

Non-parametric statistics were used in the study. Kruskal Wallis H test was used to compare body weight, seminiferous tubule diameter, and Johnsen Testicular Biopsy Scores between groups, and Mann Whitney U test was performed between paired groups for differences between significant variables. However, Wilcoxon Test was used for the difference in body weights of the groups on the 1st and 30th days. Analyses were carried out using SPSS v.22.

## Results

***Live weight findings***

There was no statistically significant difference between the groups in terms of the weight of the rats on day 1 (χ2=0.284, SD=3, *P>*0.05). When the body weights of the rats were compared between the groups, a significant difference was observed (χ2=29.856, SD=3, *P<*0.001) at day 30. It was determined that there was no statistically significant difference between the control group and the sham group (*P*=0.171), while the differences between all other paired groups were statistically significant (*P<*0.01).

The control group (z = 2.521, *P<*0.05) in the Sham group (z=2.524, *P<*0.05), 0.5 mg/kg ROF (z=2.623, *P<*0.01) and 1 mg/kg ROF (z=2.814, *P<*0.01) weights found a statistically significant difference between groups 1 and 30 days. When the change in body weight within 30 days was examined, a statistically significant increase at the end of 30 days was observed in the control group and sham group. A statistically significant decrease was observed at the end of 30 days in the 0.5 mg/kg and 1 mg/kg ROF groups (Table 4).

***Seminiferous tubule diameter***

There was a statistically significant difference between the groups in terms of the seminiferous tubule diameter of rats (χ2=23.959, SD=3, *P<*0.001). There was no statistically significant difference between the control and sham groups in terms of the seminiferous tubule diameter of rats (*P>*0.05), but it was observed that there was a statistically significant difference between the control group and 0.5 mg/kg ROF (*P<*0.05) and 1 mg/kg ROF (*P<*0.001) groups. However, there was no statistically significant difference between the sham group and the 0.5 mg/kg ROF group (*P>*0.05) (Table 5).

***Johnsen’s testicular biopsy score***

According to the Johnsen Testicular Biopsy Score, there was no significant difference between the control and sham groups (*P>*0.05), but there was a significant difference between the 0.5 mg/kg and 1 mg/kg ROF groups and the control and sham groups (*P<*0.05). Statistical analysis results according to Johnsen Testicular Biopsy Score are given in Table 6.

***Histopathological findings***

**Figure 1:**
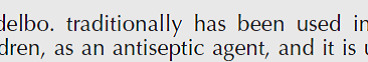
Rat testicular tissue, H&E staining. (a): Sham group, (b): 0.5 mg/kg ROF group, (c): 1 mg/kg ROF group, Seminiferous epithelium (se), Degenerate tubule (*), Interstitial edema (arrow), x40-H&E

It was observed that the seminiferous tubules and spermatogenic and Sertoli cells in these tubules were normal in the testicular tissues of the rats in the control and sham groups. Interstitial connective tissue and interstitial cells were found between the seminiferous tubules. It was observed that the full structures of the seminiferous tubules began to disappear and the normal ordered structures of the spermatogenic cells began to deteriorate in the testicular tissues of the rats in the 0.5 and 1 mg/kg ROF groups. In the 0.5 mg/kg ROF group, shedding of the seminiferous epithelium and local degenerations in the interstitial area were observed, while in the 1 mg/kg ROF group, intercellular separation, desquamation, interstitial edema, and degenerative changes were observed (Figure 1).

***Immunohistochemical findings***

**Figure 2:**
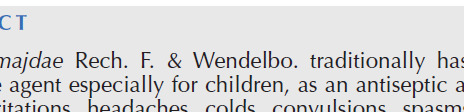
Rat testicular tissue, Immunohistochemical staining. (a): Control group Caspase-3 negativity, (b): Control group AIF negativity, (c): Control group LC3B negativity, (d): Sham group Caspase-3 negativity, (e); Sham group AIF negativity, (f): Sham group LC3B negativity, x20-IHC

Caspase-3, AIF, and LC3B immunoreactivity (Figure 2) were not statistically significant in the testicular tissues of control and sham group rats (*P>*0.05).

**Figure 3:**
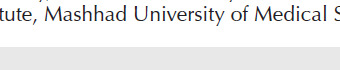
Rat testicular tissue, Immunohistochemical staining. (a): 0.5 mg/kg ROF group Caspase-3 positivity, (b): 0.5 mg/kg ROF group AIF positivity, (c): 0.5 mg/kg ROF group LC3B positivity, (d): 1 mg/kg ROF group Caspase-3 positivity, (e): 1 mg/kg ROF group AIF positivity, (f): 1 mg/kg ROF group LC3B positivity (arrow), x20-IHC

Mild Caspase-3, AIF, and LC3B immunoreactivity were observed in the interstitial area in testicular tissues of rats in the 0.5 mg/kg ROF group, while mild LC3B immunoreactivity was also observed in the seminiferous epithelium (Figure 3). Caspase-3 and AIF immunoreactivity were mild in the interstitial area, and LC3B immunoreactivity was severe in the interstitial area and seminiferous epithelium in testicular tissues of rats in the 1 mg/kg ROF group (Figure 3). There was a statistically significant difference between the control and sham groups and the 0.5 and 1 mg/kg ROF groups (Table 7, *P<*0.05).

***Immunofluorescence findings***

**Figure 4:**
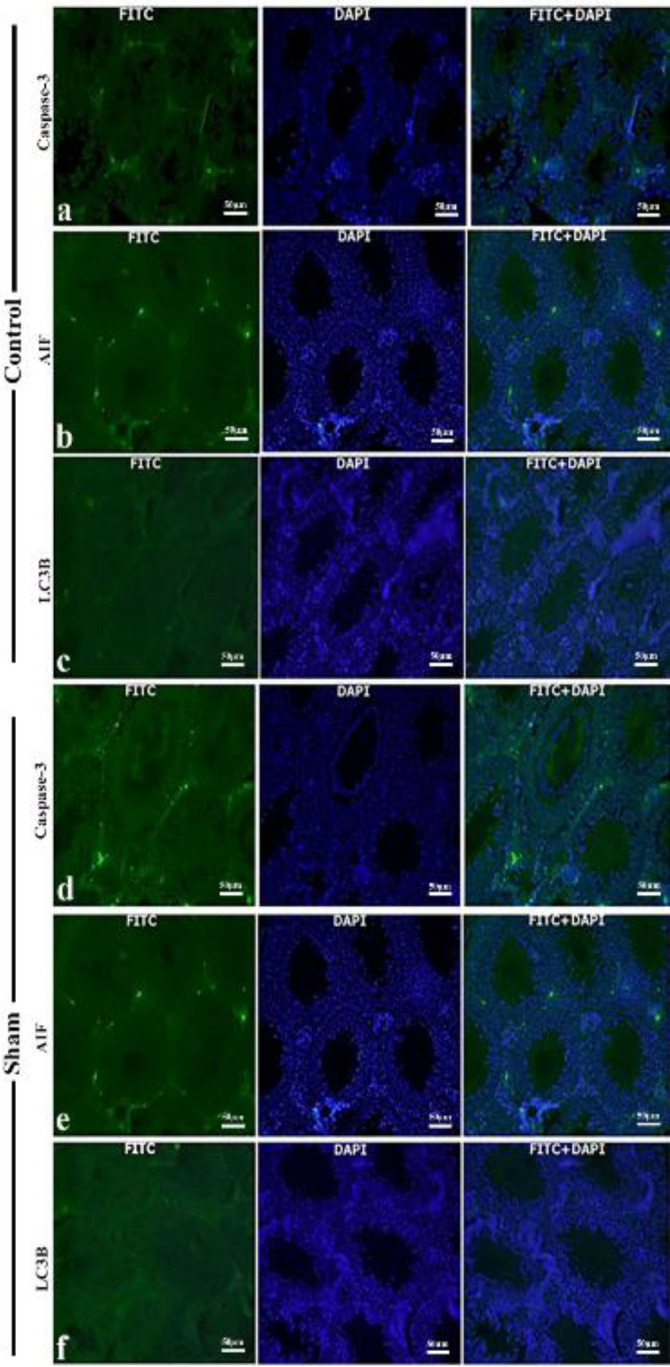
Rat testicular tissue, immunofluorescence staining

**Figure 5:**
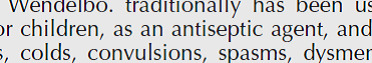
Rat testicular tissue, immunofluorescence staining

No significant difference was observed in immunopositivity in terms of Caspase-3, AIF, and LC3B in testicular tissues of rats in the control and sham groups (Figure 4), and no statistical difference (*P>*0.05). It was determined that Caspase-3, AIF, and LC3B expression levels in testicular tissues of rats in the 0.5 mg/kg ROF group were increased compared with the control and sham groups and were mildly severe in the interstitial area. Caspase-3, AIF, and LC3B immunopositivity in testicular tissues of rats in the 1 mg/kg ROF group was moderate in the interstitial area (Figure 5). There was a significant difference between the control and sham groups and the 0.5 and 1 mg/kg ROF groups (Table 8, *P<*0.05).

***Biochemical findings***

In terms of serum testosterone levels, there was no statistically significant difference between the control and sham groups (*P*=0.144), the 0.5 mg/kg ROF group was lower than the control and sham groups (*P*=0.001 and *P*=0.014, respectively), and the 1 mg/kg ROF group was found to be lower compared with the control, sham, and 0.5 mg/kg ROF groups (*P*=0.000, *P*=0.000, and *P*=0.027, respectively) (Table 9).

**Table 1:** Experimental groups and applications to rats

**Groups**	**N**	**Application**
Control	8	None
Sham	8	1 ml physiological saline solution for 30 days
0.5 mg/kg ROF group	10	0.5 mg/kg roflumilast for 30 days
1 mg/kg ROF group	10	1 mg/kg roflumilast for 30 days

ROF: roflumilast

**Table 2:** Primary antibody information used in histologic analyses

Primary antibody	Company and catalog no.	Dilution rate	Duration and temperature
**Cleaved ** **– ** **Caspase-3** ^† ^	Cell Signaling Company, catalog no. 9661	1/200	20 min at room temperature
**AIF ** **– ** **1 polyclonal** ^‡^	Aviva Systems Biology, catalog no. OAGA04280	1/200	20 min at room temperature
**LC3B polyclonal** ^⁋^	Abclonal, catalog no. A7198	1/200	20 min at room temperature

† Used to determine caspase-dependent apoptosis; ‡ Caspase was used to determine independent apoptosis; ⁋ Used to determine autophagy

**Table 3:** Fluorescence-induced secondary antibody information used in Immunofluorescence staining

Secondary antibody	Company, catalog no.
**Goat Anti-Mouse IgG- FITC**	Jackson ImmunoResearch, Catalog No. 115-095-003
**Mouse Anti-Rabbit IgG-FITC**	Santa Cruz, Catalog No. sc-2359

**Table 4:** Live weight values of rats and comparison of these values

Groups	1st Live weight - g/day mean ± SD (Min-Max)		30th Live weight - g/day mean ± SD (Min-Max)		*P *(1st vs 30th)
**Control (n=8)**	193.00 ± 5.80 (185.00 – 200.00)		210.50 ± 9.05 (197.00 – 220.00)		0.012**
**Sham (n=8)**	192.63 ± 4.17 (187.00 – 200.00)		204.50 ± 7.70 (195.00 – 218.00)		0.012**
**0.5 mg/kg ROF Group (n=10)**	193.80 ± 5.05 (185.00 – 202.00)		190.70 ± 4.66 (184.00 – 201.00)		0.009**
**1 mg/kg ROF Group (n=10)**	192.80 ± 4.78 (187.00 – 200.00)		179.60 ± 2.06 (176.00 – 182.00)		0.005**
1st Day live weight group comparisons					
Groups				***P***	
**Control **–** Sham **				0.915	
**Control **–** 0.5 mg/kg ROF Group**				0.859	
**Control **–** 1 mg/kg ROF Group**				1.000	
**Sham **–** 0.5 mg/kg ROF Group**				0.624	
**Sham **–** 1 mg/kg ROF Group**				1.000	
**0.5 mg/kg ROF Group **–** 1 mg/kg ROF Group**				0.595	
30th Day live weight group comparisons					
Groups				***P***	
**Control **– **Sham **				0.171	
**Control **–** 0.5 mg/kg ROF Group**				0.01**	
**Control **–** 1 mg/kg ROF Group**				0.000*	
**Sham **–** 0.5 mg/kg ROF Group**				0.001*	
**Sham **– **1 mg/kg ROF Group**				0.000*	
**0.5 mg/kg ROF Group **–** 1 mg/kg ROF Group**				0.000*	

*P<0.005, **P<0.05 SD: Standard Deviation; ROF: roflumilast

**Table 5:** Seminiferous tubule diameter measurement results of rats and comparison of the results

Groups	Seminiferous tubule diameter (µm) mean ± SD (Min–Max)
**Control (n=8)**	281.88 ± 1.72 (279.00 – 284.00)
**Sham (n=8)**	281.00 ± 0.756 (280.00 – 282.00)
**0.5 mg/kg ROF Group (n=10)**	280.20 ± 0.789 (279.00 – 281.00)
**1 mg/kg ROF Group (n=10)**	275.40 ± 2.95 (271.00 – 279.00)
Seminiferous tubule diameter group comparisons	
Groups	***P*** **-value**
**Control **–** Sham **	0.197
**Control **–** 0.5 mg/kg ROF Group**	0.036**
**Control **–** 1 mg/kg ROF Group**	0.000*
**Sham **–** 0.5 mg/kg ROF Group**	0.057
**Sham **–** 1 mg/kg ROF Group**	0.000*
**0.5 mg/kg ROF Group **–** 1 mg/kg ROF Group**	0.000*

*P<0.005, **P<0.05 SD: Standard Deviation; ROF: roflumilast

**Table 6:** The results of Johnsen testicular biopsy of rats in groups

Groups	Johnsen testicular biopsy score mean ± SD
**Control**	9.50 ± 0.53^a^
**Sham**	9.37 ± 0.51^a^
**0.5 mg/kg ROF**	7.37 ± 0.18^b^
**1 mg/kg ROF**	7.50 ± 0.18^b^

^a, b^ Different letters in the same column show the difference between groups

SD: Standard Deviation; ROF: roflumilast

**Table 7:** Statistical representation of immunohistochemical staining in terms of caspase-3, AIF, and LC3B

Groups	Caspase-3 mean ± SD	AIF mean ± SD	LC3B mean ± SD
**Control**	0.25 ± 0.46^Aa^	0.37 ± 0.51^Aa^	0.25 ± 0.46^Aa^
**Sham**	0.12 ± 0.35^Aa^	0.37 ± 0.51^Aa^	0.25 ± 0.46^Aa^
**0.5 mg/kg ROF**	1.12 ± 0.64^Ba^	1.25 ± 0.70^Ba^	2.12 ± 0.35^Bb^
**1 mg/kg ROF**	2.12 ± 0.35^Ca^	2.00 ± 0.53^Ca^	2.87 ± 0.35^Cb^

^a,b,c^ Different letters in the same column show the difference between groups, ^A, B^ Different letters in the same line indicate the difference in the groups

SD: Standard Deviation; ROF: roflumilast

**Table 8:** Statistical representation of immunofluorescence staining in terms of caspase-3, AIF, and LC3B

Groups	Caspase-3 mean ± SD	AIF mean ± SD	LC3B mean ± SD
**Control**	0.50 ± 0.53^Aa^	0.37 ± 0.51^Aa^	0.50 ± 0.53^Aa^
**Sham**	0.62 ± 0.51^Aa^	0.50 ± 0.53^Aa^	0.75 ± 0.46^Aa^
**0.5 mg/kg ROF**	1.75 ± 0.46^Bb^	1.25 ± 0.70^Bb^	1.87 ± 0.35^Bb^
**1 mg/kg ROF**	1.87 ± 0.35^Bb^	1.75 ± 0.46^Bb^	2.00 ± 0.53^Bb^

^a, b^ Different letters in the same column show the difference between groups, ^A, B^ Different letters in the same line indicate the difference in the groups

SD: Standard Deviation; ROF: roflumilast

(a): Control group Caspase-3 immunoreactivities, (b): Control group AIF immunoreactivities, (c): Control group LC3B immunoreactivities, (d): Sham group Caspase-3 immunoreactivities, (e): Sham group AIF immunoreactivities, (f): Sham group LC3B immunoreactivities, x20-IF

(a): 0.5 mg/kg ROF group, Caspase-3 immunoreactivities, (b): 0.5 mg/kg ROF group, AIF immunoreactivities, (c): 0.5 mg/kg ROF group, LC3B immunoreactivities, (d): 1 mg/kg ROF group, Caspase-3 immunoreactivities, (e): 1 mg/kg ROF group, AIF immunoreactivities, (f): 1 mg/kg ROF group, LC3B immunoreactivities. Interstitial area (➤)x20-IF

**Table 9:** Serum testosterone level results of rats in groups

Groups	Serum testosterone level (ng/ml) mean ± SD	
**Control**		0.48 ± 0.01^ a^
**Sham**		0.46 ± 0.02^ a^
**0.5 mg/kg ROF**		0.40 ± 0.05^ b^
**1 mg/kg ROF **		0.35 ± 0.03^ b^

^a, b^ Different letters in the same column show the difference between groups

SD: Standard Deviation; ROF: roflumilast

## Discussion

ROF is the only PDE4 inhibitor that reaches the market for patients with chronic bronchitis symptoms and COPD (22). Several new PDE4 inhibitor compounds are in early clinical development, and there is not yet clear information about their efficacy and safety (23). In preclinical and early-stage studies, it has been reported that PDE4 inhibitors improve memory and have curative effects on depressive-like behaviors caused by chronic mild stress, lipopolysaccharide, or ethanol abstinence (24, 25). ROF has been studied by many researchers to determine the mechanism and new indications. ROF 5-10 mg/kg was administered orally to mice once a day for 30 days. It has been reported that ROF has anti-apoptotic and anti-inflammatory effects on nerve cells, as well as being an effective agent in the treatment of depression and memory disorders (26). In cadmium (Cd) derived nephrotoxic rats, they reported a significant increase in anti-oxidative enzymes such as Superoxide Dismutase, Catalase, and Glutathione-S-transferase, and a decrease in oxidative parameters such as Malondialdehyde and ischemic modified albumin. However, it has been reported that the damage caused by Cd in kidney tissues significantly recurs from ROF treatment (0.5 and 1.5 mg/kg) (27).

It has been asserted that ROF, which is used orally as 500 µg tablets, once daily, does not cause arrhythmia and the most common side effects are diarrhea and weight loss (28). It has been reported that weight loss, one of the most important side effects of ROF, was observed in 83 adult COPD cases because of treatment with ROF (500 µg tablet, 18 months, once a day) (29). We found that while an increase in rat body weight was observed in the control and sham groups in accordance with the months, the body weights of the rats exposed to ROF decreased as the dose increased in accordance with the literature.

It has been reported that the dose and duration of application of phosphodiesterase inhibitors are determinative for efficacy, but long-term use at high doses causes adverse effects (30). In our study, in the 0.5 mg/kg ROF group, the testicular tissue of the rats was narrowed in seminiferous tubule diameter and degeneration was observed in the interstitial area, while the damage became clearer in the 1 mg/kg ROF group. It was observed that the narrowing of the tubule diameter was statistically significant. The separation between cells, desquamation, degenerative changes, and edematous areas in the interstitial connective tissue were observed.

It has been reported that PDE4 increases cell viability and mitochondrial activity, and decreases cell death (31, 32). It has been asserted that PDE4 may be a new therapeutic agent in liver fibrosis, increasing the level of cAMP, which suppresses collagen synthesis and fibroblast activation (33). The effects of ROF on cerebral inflammation developing in the subarachnoid hemorrhage model in rats were investigated and it was shown that it significantly reduced neurological damage and inflammatory cytokines IL1β, IL-6, and TNFα levels and the number of apoptotic neurons (34). The cerebral cortices of rats treated with ROF were examined by the TUNEL method and it was determined that ROF administration significantly reduced apoptosis (26). In a study in which a sepsis model was created by cecal ligation and puncture surgery, ROF (1 mg/kg and 3 mg/kg) was applied to mice once a day for 7 days, and it was concluded that 3 mg/kg ROF could protect mice against kidney damage by partially reducing cell apoptosis (35). In the study, in which the PDE4 enzyme inhibitor rolipram was applied to rats (single dose 10 mg/kg) 15 min before detorsion and the effects on histopathological damage after testicular torsion/detorsion and the apoptotic pathways thought to cause damage; it was found that rolipram could not reverse histological damage, suppress the activated intrinsic apoptotic pathway, and did not have a protective effect against histopathological damage (36). We determined that caspase-3 expression increased in healthy testicular tissue, especially at high doses, in chronic use of ROF in line with the results of this study.

Many studies of male reproduction in the cAMP with changes in sperm motility and acrosome reaction hiperaktivasyon capable of entering into the scheme of improvement and that is required for events that occur during kapasitasyon notified when cAMP levels control because it did not increase in the case of hyper-cellular processes, including proliferation can cause emphasize and also create problems in the formation of cancer (37-40). It has been stated that while apoptosis that occurs under physiological conditions in the immature testis is necessary for the development of germ cells, misactivation of apoptosis may impair spermatogenesis and cause reproductive defects (41, 42). In the results of this study, it was observed that the intensity of Caspase-3 and AIF immunoreactivity, which are important apoptosis reagents in spermatogenesis, increased more in rat testis tissue in the 1 mg/kg roflumilast group compared with 0.5 mg/kg roflumilast group. Research related to the subject shows that chronic and high doses of the use of ROF may affect the spermatogenesis process negatively.

Autophagy activation has been shown to play a regulatory role in the maintenance of spermatogenesis and the maintenance of spermatogenic stem cells. When the activation of LC3-2, LC3-2/LC3-1, Atg5, and Beclin-1 autophagic reagents were examined by spermatogenic stem cell culture study of organophosphate, which is toxic for the reproductive system; it was reported that all these reagents are increased in rat spermatogenic stem cells (43). In the study investigating the role of autophagy in testicular tissue, germ cell-specific Atg7 deletion was performed and the fertility of Atg7 mice was evaluated. It has been observed that there are very few sperms in the epididymis, most of the sperms exhibit round head anomaly, and the acrosome structure is damaged (44). In this study, we found that LC3B expression increased in rats exposed to 0.5 and 1 mg/kg ROF for 4 weeks, and there was positivity in some spermatogenic cells, unlike caspase-3 and AIF. Based on these findings, it was thought that structural and functional changes in testicular tissue might be associated with increased autophagy.

Testosterone is an important androgen in the development of the male reproductive system and sexual characteristics. It has been reported that testosterone regulates the cGMP pathway and thus affects endothelial function and endothelial progenitor cells, which are key to the endothelial repair system (45). It has been asserted that decreased testosterone level causes structural and functional changes in Sertoli cells, which are important for germ cells (46). After long-term treatment with sildenafil, a PDE5 inhibitor; it has been reported that the circulating testosterone level is normalized and contributes to the accumulation of cGMP in the testicular interstitial fluid, resulting in an improvement in Leydig cell steroidogenic capacity and an increase in the number of Leydig cells, in addition to preventing the atrophy of the seminiferous tubules (47). The inhibition of PDE4 enzymes in testosterone production by the Leydig cells has been reported to cause a significant increase. In rats, it has been asserted that autophagy participates in the regulation of steroid synthesis in Leydig cells (48). It has been reported that PDE4 and PDE8 play important roles in the physiology of steroidogenic tissues and there is a synergism between signaling compartments regulated by PDE4 and PDE8 to facilitate maximum steroid output (49). It has been asserted that the repository of cAMP, which regulates androgen production, is controlled by PDE8s working together with PDE4, and that PDE inhibitor therapy should simultaneously target both PDE8 isozymes and PDE4 to be an effective stimulator of steroidogenesis (50). In our findings, it was revealed by the ELISA method that the serum testosterone level was within the normal reference range in the control and sham groups (*P*=0.001 and *P*=0.014), but with the increase in the dose used in the roflumilast groups, the testosterone level decreased statistically significantly (*P*=0.000, *P*=0.000, *P*=0.027).

## Conclusion

It is considered that degenerated and narrowed seminiferous tubule structures, increased caspase-3, AIF, and LC3B immunoreactivity density, and decreased serum testosterone levels with increasing dose. ROF may have some anabolic effects and have negative effects on spermatogenesis and therefore male fertility due to changes in the mechanism of apoptosis and autophagy in this study. However, more detailed experimental studies and clinical findings are needed to determine the toxicities that may occur in chronic use of high doses of ROF.

## Authors’ Contributions

AG and EKS designed and performed the research, analyzed data, and wrote and edited the article.

## Conflicts of Interest

The authors of this manuscript have no conflicts of interest to declare.
